# A Flexible Temperature Sensor Integrated at Needle Tip for In Situ Acupoint Temperature Monitoring

**DOI:** 10.3390/mi15070924

**Published:** 2024-07-19

**Authors:** Ci Song, Zheng Yu, Weiwen Feng, Ke Sun, Chuanbiao Wen, Shengyan Zhang, Shuguang Yu, Xinxin Li

**Affiliations:** 1 State Key Laboratory of Transducer Technology, Shanghai Institute of Microsystem and Information Technology, Chinese Academy of Sciences, Shanghai 200050, China; songci@mail.sim.ac.cn (C.S.); fengww@mail.sim.ac.cn (W.F.); sunke@mail.sim.ac.cn (K.S.); 2 University of Chinese Academy of Sciences, Beijing 100049, China; 3 School of Intelligent Medicine, Chengdu University of Traditional Chinese Medicine, Chengdu 611137, China; yuzheng@cdutcm.edu.cn (Z.Y.); wenchuanbiao@cdutcm.edu (C.W.); 4 Acupuncture and Tuina School, Chengdu University of Traditional Chinese Medicine, Chengdu 611137, China; zhangshengyan@stu.cdutcm.edu.cn (S.Z.)

**Keywords:** MEMS manufacturing technology, flexible, temperature sensor, thermistor, acupoint temperature

## Abstract

Temperature can reflect vital activities, and researchers have attempted to guide Chinese medicine diagnosis and treatment by observing acupoint temperature changes. Integrating a temperature sensor at the needle tip enables in situ acupoint temperature measurement. However, the sensor needles for acupoint temperature monitoring designed in previous studies were fabricated by manually soldering thermistor beads and metal wires, making mass production difficult. In this work, using MEMS manufacturing technology, a flexible temperature sensor that can be integrated at the needle tip is proposed and can be mass-produced on silicon wafers. The sensor uses a Pt thermistor as the temperature-sensing element and has a slender flexible structure with dimensions of 125 μm width by 3.2 cm length. As the sensor is inserted into a hollow needle, the Pt thermistor is glued to the needle tip. In the temperature range of 30 °C to 50 °C, the fabricated temperature sensor has a sensitivity of 5.00 Ω∙°C^−1^, a nonlinearity of ±0.39%FS, and a repeatability error of ±2.62%FS. Additionally, the sensor has been applied to in vivo acupoint temperature monitoring experiments in rats and demonstrated good performance, suggesting its promise for future research on acupoint temperature.

## 1. Introduction

Temperature is an important indicator of life activity, and temperature measurement is one of the most important ways to reveal vital signs. Temperature changes can reflect the material metabolism and energy transformation of an organism. However, body surface temperature is susceptible to ambient temperature, and the temperature of the underlying tissues reflects what is going on in the tissues of living organisms better. Therefore, measuring subcutaneous temperature is more informative compared to measuring temperature on the skin.

Acupuncture is a medical treatment in traditional Chinese medicine. It involves inserting one or multiple fine metal needles into the skin and underlying tissues at precise points on the body. These precise points are called acupoints. In addition to the traditional diagnostic methods of acupoints, modern researchers have explored measuring acupoint temperature and relating it to anatomy, which can reveal physiological and pathological changes in the human body, thus guiding clinical practice [1,2,3,4,5,6,7,8,9,10]. In a previous study, Cui et al. welded thermistor beads and metal wires together to make a temperature sensor and threaded it into hollow needles [11]. However, this process relies on manual completion, and the device performance was susceptible to the quality of the soldering, making it difficult to achieve batch fabrication. Sensors manufactured by microelectromechanical systems (MEMS) technology have the advantages of miniaturization, high integration, and mass production, gradually replacing traditional mechanical sensors.

In sensor manufacturing technology, silicon or other hard materials are generally used to fabricate rigid sensors in order to improve the mechanical strength, reliability and lifetime of the device [12,13]. However, in certain specialized measurement scenarios, such as signal sensing on the surface of irregularly shaped objects or within the interior of living organisms, flexible sensors are widely employed in medical industries, aerospace, robotics, consumer electronics and other fields [14,15,16,17]. Based on different temperature measurement principles, flexible temperature sensors can be categorized into flexible thermocouple temperature sensors and flexible resistance temperature sensors [18]. According to the Seebeck effect, the flexible thermocouple temperature sensor converts the temperature difference between the cold and hot ends of the thermocouples into an electric potential for relative temperature measurement. The temperature measurement sensitivity is determined by the difference in the Seebeck coefficients of the two materials forming the thermocouple. Flexible resistance temperature sensors utilize the dependence of resistance material on temperature for temperature measurement. Compared to thermocouple temperature sensors, resistance temperature sensors have a simpler signal readout mechanism, requiring only an accurate measurement of the resistance to obtain the corresponding temperature.

Common thermal thin-film materials for flexible resistance temperature sensors include semiconductor materials, metals, and carbon-based nanomaterials. In 2011, a flexible polycrystalline silicon temperature sensor based on polycrystalline silicon resistors and flexible SU-8 material was proposed. This temperature sensor could achieve ~0.9 °C sensing accuracy, but its fabrication process was complicated involving metal-semiconductor ohmic contacts [19]. In 2019, Cui et al. embedded silver nanowires (AgNW) within polyimide films and measured the temperature at human joints [20]. The temperature coefficient of this flexible temperature sensor had a temperature coefficient of resistance (TCR) of 0.0033 °C^−1^ and a sensitivity of 0.47 Ω∙°C^−1^. In 2020, Soni et al. used poly(3,4-ethylenedioxythiophene): poly (styr-enesulfonate) (PEDOT: PSS)-graphene oxide (GO) as a thermosensitive material and printed it on a PI substrate to fabricate a flexible temperature sensor, which achieved a TCR of 0.0109 °C^−1^. However, the fabrications of these two sensors are not compatible with standard semiconductor manufacturing techniques. Platinum (Pt) is a commonly used metal in MEMS fabrication. Due to its stable temperature coefficient over a wide temperature range and excellent film ductility, it is widely applied to flexible resistance temperature sensors [21,22,23,24,25].

Using MEMS manufacturing technology, the flexible temperature sensor developed in this paper is based on the thermal properties of the Pt resistor, which has a slender structure and can be packaged in a hollow needle with an outer diameter of 300 μm. The sensor has been proven to exhibit high sensitivity, strong linearity, and satisfactory repeatability, thereby providing a reliable measurement tool for the study of acupoint temperature. And it has been applied to in vivo acupoint temperature measurement in rats. Additionally, the device can also be used as a microheater for localized heating of subcutaneous tissue.

## 2. Design

**Figure 1:**
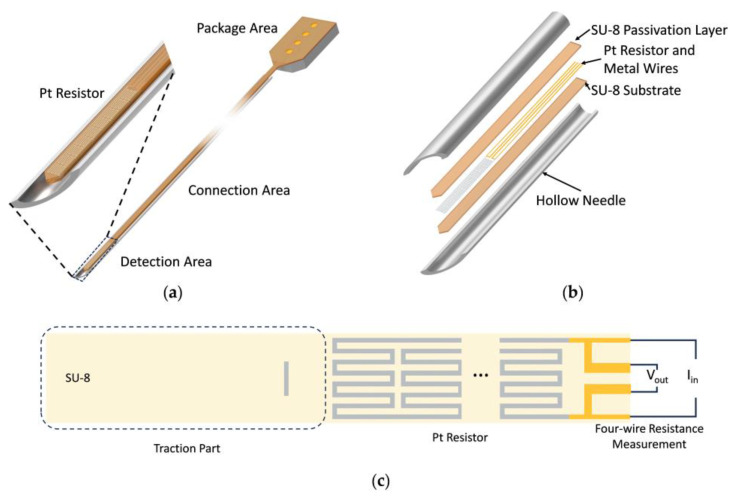
Structure design of the flexible temperature sensor. (**a**) Overall schematic diagram. (**b**) Structure of each layer in the front of the device. (**c**) Details of flexible structure design.

The overall structure of the flexible temperature sensor is illustrated in Figure 1a. The sensor consists of three main components: the detection area, connection area, and package area. In the detection area, a thin-film Pt resistor is fabricated on the flexible substrate to serve as the temperature-sensing element. In the connection area, four metal leads connect the Pt resistor to four metal pads in the package area, where the silicon substrate is retained for the convenience of bonding the sensor to an external system. The front flexible part with a width of 125 μm and a length of 3.2 cm will be threaded into the needle tube, and the detection area will be fixed at the needle tip for in situ acupoint temperature monitoring.

Figure 1b shows the structure of each layer in the front of the device. The polymer SU-8 is used to construct the flexible substrate and to be the passivation layer for electrical insulation. The Pt resistor and metal wires are located in the middle of two SU-8 layers. As shown in Figure 1c, an additional section of SU-8 with a length of 0.2 cm is designed at the front of the sensor as the traction part, which helps thread the slender sensor into the hollow needle. Four-wire resistance measurement is to place a known current flow through the Pt resistor and measure the voltage drop across it, which eliminates errors caused by the resistance of the leads to the resistor [26]. 

To ensure high sensitivity and linearity of the sensor, we discussed the thickness of the thin-film Pt resistor. Platinum resistors have a wide temperature test range of −200 °C to 850 °C. The temperature resistance relationships of Pt resistors are expressed as follows [27]:

(1) $$R_{T}=\left\{\begin{array}{ll} {\left[1+\alpha_{1}T+\alpha_{2}T^{2}+\alpha_{3}T^{3}\left(T-100\right)\right]R}_{T_{0}}, & -200\;{}^{\circ}C\leq T<0\;{}^{\circ}C\\ {\left(1+\alpha_{1}T+\alpha_{2}T^{2}\right)R}_{T_{0}\;}, & 0\;{}^{\circ}C\leq T<850\;{}^{\circ}C \end{array}\right.$$

where $R_{T}$ is the resistance at a temperature of *T*, $R_{T_{0}}$ is the resistance at an initial temperature of *T*_0_, and *α*_1_, *α*_2_, and *α*_3_ are the temperature coefficients of resistance (TCR). Since the sensor is used for acupoint temperature measurements, only the case where the temperature is greater than 0 °C is discussed. The first-order temperature coefficient of platinum is much larger than the second-order temperature coefficient, so the temperature sensitivity of Pt can be calculated as

(2) $$S_{T}=\frac{dR_{T}}{dT}\approx \alpha_{1}R_{T_{0}}$$

**Figure 2:**
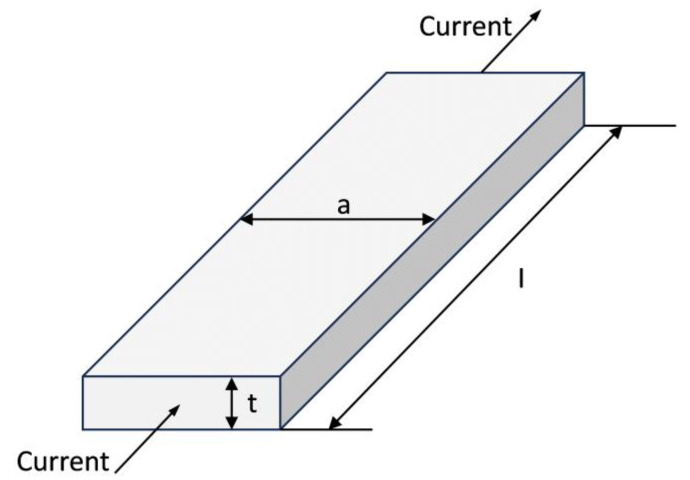
Schematic diagram of the equivalent platinum resistor.

The schematic diagram of the equivalent platinum resistor is shown in Figure 2. When the current as shown flows through the resistor, the resistance can be expressed as

(3) $$R_{T_{0}}=\rho \cdot \frac{l}{a\cdot t}$$

where ρ is the resistivity of the platinum resistor, l is the length of the resistor, a is the width of the cross-sectional area of the resistor, and t is the thickness of the resistor. Therefore, the temperature sensitivity of Pt can be rewritten as

(4) $$S_{T}=\alpha_{1}\cdot \rho \cdot \frac{l}{a\cdot t}$$

According to Equation (4), the higher sensitivity requires a smaller resistor thickness. However, the resistor properties of thin films with sufficiently small thicknesses differ from those of bulk materials, and the film thickness may affect the resistivity ρ and temperature coefficient *α*. According to Matthiessen’s rule, the net resistivity of a metal is the sum of the resistivities caused by surface electron scattering, grain boundary scattering, phonon scattering, defects, and impurities, and it can be expressed as [28]

(5) $$\rho =\rho_{s}+\rho_{g}+\rho_{p}+\rho_{d}+\rho_{i}$$

When the resistor is in bulk form, the resistivity is an intrinsic property of the material, independent of its shape or size. As the thickness of the metal film decreases, the probability of surface electron scatter increases. When the thickness gradually decreases to the electron mean free path *λ*, the surface electron scatter tends to diffuse scatter, and the resistivity $\rho_{s}$ plays the main role in affecting the net resistivity ρ [29,30,31,32,33]. In this case, the resistivity of the metal film is related to the shape and thickness. When the ratio of the metal film thickness *t* to the electron mean free path is greater than 10, the resistivity of the thin-film metal resistor tends to the bulk resistivity and is not affected by the shape of the metal [34].

Since the temperature sensor designed in this paper is flexible, its Pt resistor will deform along with the flexible substrate. To increase the thermal stability of the platinum resistance temperature sensor, we should reduce the influence of metal shape change on the sensor performance. Thus, the thickness of the platinum resistance should be 10 times greater than its electron mean free path. The electron mean free path of platinum is about 11.5 nm [35], so the minimum thickness of the platinum resistor should be more than 115 nm. In this paper, the thickness of the platinum resistor is designed as *t* = 120 nm.

To balance the high initial resistance and the difficulty of fabrication, the minimum line width of the Pt resistor is designed as *a* = 6 μm. The Pt resistor winding method shown in Figure 1c increases the total length *l* of the Pt resistor by several serpentine bends, thus increasing the initial resistance. 

**Figure 3:**
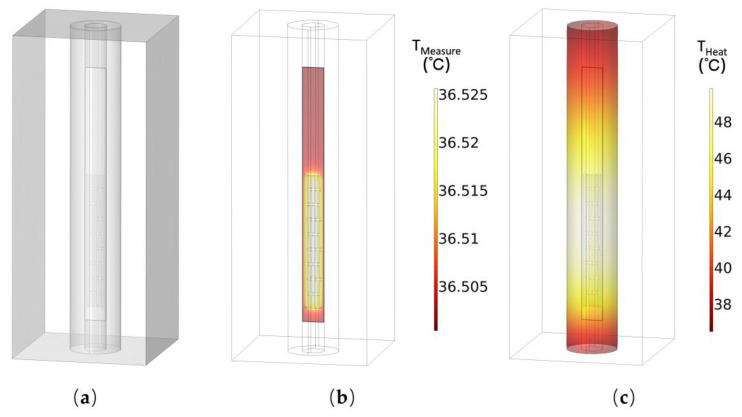
Temperature sensor finite-element simulation. (**a**) Sensor simulation model. (**b**) Finite-element simulation result of temperature distribution at the sensor surface in temperature measurement mode. (**c**) Finite-element simulation result of temperature distribution at the needle surface in heating mode.

As shown in Figure 3, the finite-element simulations are performed to validate the operation of the flexible temperature sensor by using COMSOL Multiphysics 6.0 software, where a 1:1 3D model of a flexible sensor encapsulated into a hollow needle is constructed and conjugate heat transfer is considered in this model to simulate the heat transfer of the sensor and the surrounding liquid environment. Moreover, the laminar flow field is set to simulate the natural convection of blood and tissue fluids, which refers to the flow caused only by the unevenness of the fluid’s own temperature field without relying on any external forces. Electric Currents in Layered Shells are added to define the power supply of the sensor. The Pt resistor is set as the conductive shell in Electric Currents in Layered Shells, and terminals are added in it so that a power supply can be added to the Pt resistor.

The initial temperature of the liquid and laminar flow field is set to 36.5 °C, and the simulation results are shown in Figure 3b,c. In temperature measurement mode, under the current supply of 0.1 mA, the maximum temperature rise on the thermistor surface is only 0.025 °C which is acceptable for acupoint temperature monitoring. Additionally, in heating mode, under a power supply of 140 mW, the temperature on the needle tip surface can be heated up to 50 °C, so that the sensor can be used as a microheater to heat the subcutaneous tissue for therapeutic purposes.

## 3. Fabrication Process

**Figure 4:**
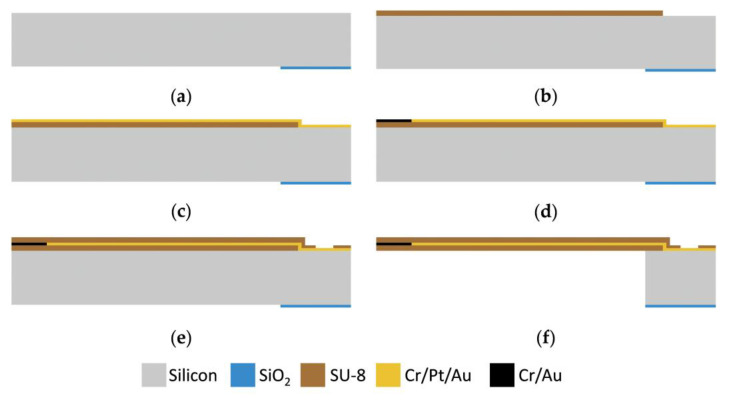
Fabrication process of the flexible temperature sensor. (**a**) Deposit and pattern SiO_2_ mask. (**b**) Fabricate flexible SU-8 substrate. (**c**) Sputter and pattern Cr/Pt/Au. (**d**) Remove Au in the temperature-sensing area by wet-etching. (**e**) Fabricate SU-8 passivation layer. (**f**) Release sensors by back etching.

According to Figure 4, the fabrication process for flexible temperature sensors contains six main steps. Unlike traditional flexible-sensor fabrication methods, this approach does not require preset adhesives or sacrificial layers on the wafer surface. Instead, the flexible sensors are fabricated directly on the wafer surface, and devices are released by back-etching the silicon-based substrate. This fabrication method conforms to standard MEMS fabrication processes, which reduces process costs and improves device stability and reliability. 

(a) After a standard wafer-cleaning process, the four-inch-diameter silicon wafer is thermally oxidized to grow a 2 μm thick SiO_2_ film on its surface. The SiO_2_ film on the front side of the wafer is removed by wet-etching with a BOE solution, while the SiO_2_ film on the back side is patterned by using the photoresist as a mask. This backside SiO_2_ film tends to be a hard mask for the final backside-etching of monocrystalline silicon.

(b) SU-8 3005 (MicroChem Co., Ltd., Adel, GA, USA) is used as the flexible structure material for this sensor, because it can be photolithographic-patterned and has high thermal stability, high chemical stability, and strong adhesion to silicon and metal [36]. A 5 μm thick SU-8 thin film is spin-coated and patterned on the wafer surface as a flexible substrate. To improve film uniformity and reduce residual stresses in SU-8 thin film, the SU-8 polymer is first spin-coated at a low speed of 500 rpm to undergo a relaxation period and then the speed is increased to 3500 rpm to achieve a thickness value of 5 μm. In addition, during post-exposure baking, a low rate of temperature rise and a multi-step baking process are used to reduce thermal shock, thus improving the mechanical properties of SU-8 films.

(c) The sensor adopts Pt as the thermistor, Cr as the adhesive layer, and Au is made on Cr/Pt as the metal leads because of its small resistivity. Cr/Pt/Au with a thickness of 40 nm/120 nm/300 nm is firstly fabricated by magnetron sputtering and then patterned by a lift-off process. The designed thermistor pattern has a minimum wire width of 6 μm and a minimum wire spacing of 8 μm which requires a special lift-off method that is superior in terms of resolution, removal, and yield. Therefore, a bi-layer lift-off process is adopted. First, a non-photosensitive material, LOR-5B (MicroChem Co., Ltd., Adel, GA, USA), is spin-coated onto the silicon wafer, and then a negative photoresist, ROL-7133 (Suzhou Research Materials Microtech Co., Ltd., Suzhou, China), is spin-coated and patterned by photolithography. During the development process, at the region where the metal needs to be retained, once the unexposed ROL-7133 is dissolved by the developer, the underlying LOR-5B is rapidly dissolved. This results in the formation of a double-layered adhesive sidewall structure with a concave lower layer. Due to the shadow effect of sputtering, this structure avoids metal sputtering on the LOR-5B sidewalls. With this method, the NMP solution can easily dissolve LOR-5B to lift off the metal above it.

(d) Using the photoresist as a mask, the Au on the surface of the Pt resistor is removed by the gold (Au)-etching solution. After removing the photoresist, the Pt resistor and metal leads are completed. It is worth noting that to reduce the risk of separation of the gold pads from the device package substrate at a high temperature during gold-wire bonding, the substrate of gold pads in the package area is monocrystalline silicon rather than flexible polymer material.

(e) Another 5 μm thick SU-8 film is fabricated on the device surface, as a passivation layer to insulate the Pt resistor and metal leads electrically.

(f) Using the SiO_2_ hard mask fabricated in step (a), the release of the device is accomplished by a special two-step backside-etching process. The first step is to etch the silicon of 350 μm in the device release area by using deep-reactive-ion-etching (DRIE). And then, the remaining 70 μm of silicon is etched by XeF_2_ vapor, which allows the device to be released in a gaseous environment at room temperature, reducing the damage rate of the flexible device.

**Figure 5:**
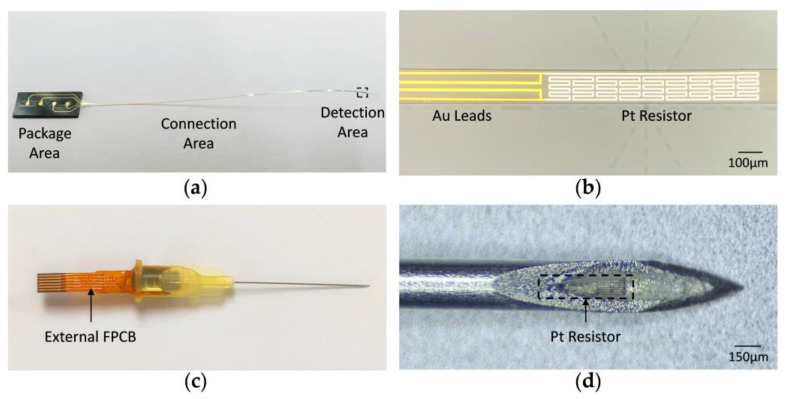
Optical images of the fabricated device. (**a**) Overall appearance of the flexible sensor. (**b**) Detailed view of the front end of the sensor. (**c**) Overall appearance of the packaged sensor needle. (**d**) Detailed view of the tip of the packaged sensor needle.

The overall appearance of the flexible sensor is illustrated in Figure 5a. Figure 5b shows the Pt resistor and Au leads fabricated by the bi-layer lift-off process. The syringe needles (300 μm outer diameter) are used as the encapsulation structures for the flexible sensor, and an external FPCB is electrically connected to the flexible sensor through gold-wire bonding. The whole temperature sensor needle is shown in Figure 5c. As is depicted in Figure 5d, the Pt resistor is fixed to the needle tip by a one-component, (UV) visible light curable silicone adhesive (LOCTITE^®^ SI 5056™, Henkel AG & Co. KGaA, Düsseldorf, Germany) which is designed for use in the assembly of disposable medical devices.

## 4. Test

### 4.1. Performance Test

**Figure 6:**
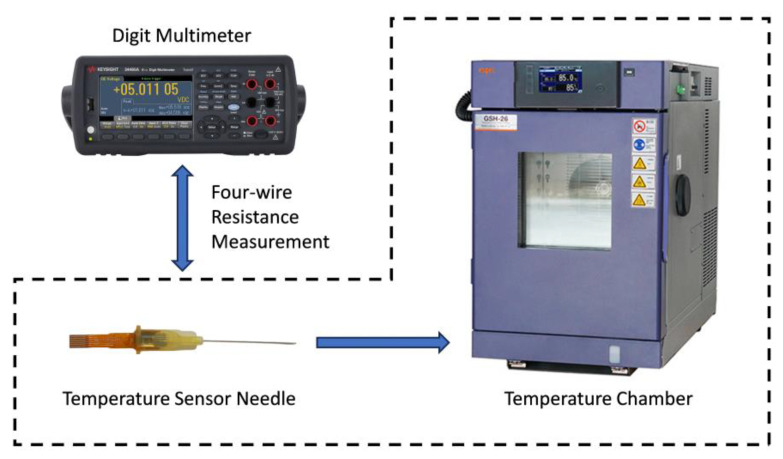
Schematic of the performance test system.

As it is shown in Figure 6, a performance test system is built for the temperature sensor. We put the packaged temperature sensor into a bench-top-type temperature chamber (GSH-26, ESPEC Co., Ltd., Osaka, Japan) and connected it to the HI and LO sense terminals of the digital multimeter (34465A, Keysight Co., Ltd., Kimballton, IA, USA) according to four-wire resistance measurement. At a room temperature of 25 °C, the resistance of the Pt thermistor is 2.35 kΩ. At a range of 10 kΩ, the standard test current of the digit multimeter is 0.1 mA, which does not cause significant heating of the sensor.

**Figure 7:**
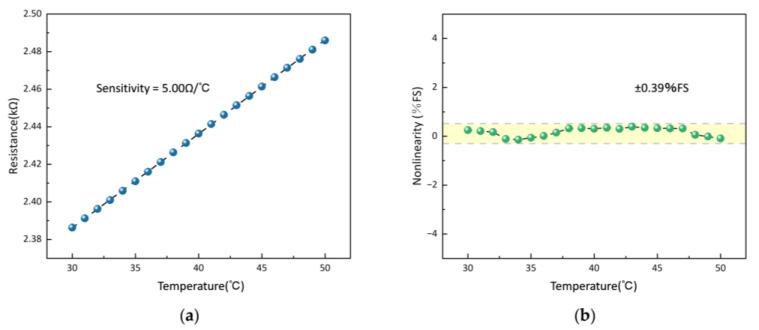
Results of the performance test. (**a**) Response curve of the temperature sensor. (**b**) Nonlinearity of temperature sensor.

Since the temperature sensor is used for acupoint temperature monitoring, we adjusted the temperature in the temperature chamber and observed the change in resistance of the Pt thermistor in the range of 30 °C to 50 °C. The results are shown in Figure 7. The sensitivity of the sensor is 5.00 Ω∙°C^−1^, and the nonlinearity of the sensor in different temperatures is within ±0.39%FS.

**Figure 8:**
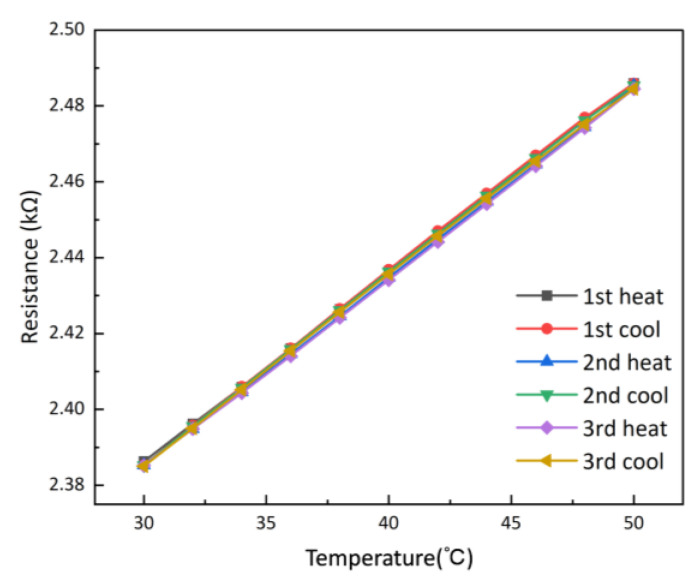
Repeatability curves of the temperature sensor.

Repeatability reflects the random error of the sensor as well as its reliability. The temperature sensor was repeatedly heated up and cooled down three times in the range of 30 °C to 50 °C using the temperature chamber. And the resistance values of the sensor corresponding to different temperatures were recorded after a 5 min stabilization period. The repeatability curve is shown in Figure 8, and the repeatability error is ±2.62%FS.

### 4.2. In Vivo In Situ Acupoint Temperature Monitoring in Rats

To validate the performance of the sensor in vivo, several experiments were designed to observe the temperature changes in the Zusanli point before and after electro-acupuncture (EA), using four healthy rats as the experimental subjects. The Zusanli (ST36) point is located approximately 5 mm lateral to the knee joint [37]. All rats used for the experiments were purchased from Chengdu Dashuo Experimental Animal Co., Ltd. (Chengdu, China) and housed in the Animal Experimental Center of the Chengdu University of Traditional Chinese Medicine. All animal-related experiments were approved by the Animal Ethics Committee of Chengdu University of TCM (receipt number: 2023354; approval date: 12 December 2023).

**Figure 9:**
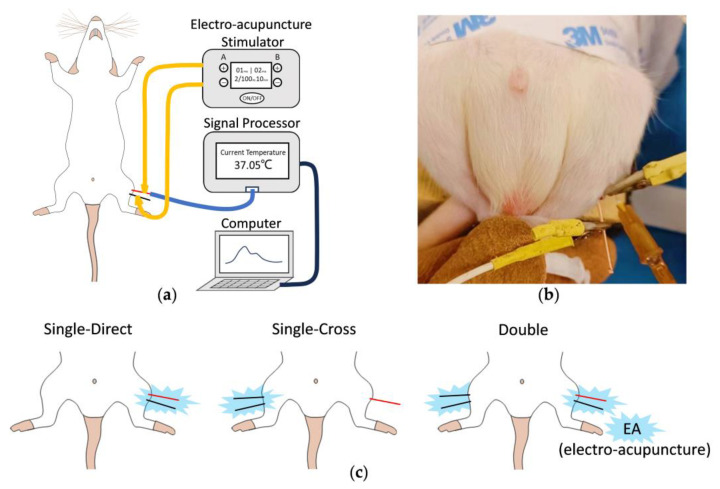
In vivo in situ acupoint temperature monitoring in rats (red lines indicate the temperature sensor needles; black lines indicate regular acupuncture needles). (**a**) Schematic diagram of in situ acupoint temperature monitoring system. (**b**) Photo-documenting the operation of in situ acupoint temperature monitoring in vivo. (**c**) Schematic diagram of EA experiments.

The in situ acupoint temperature monitoring system is shown in Figure 9a. We built a signal processor with a microcontroller (ESP32-S3) and an external screen, which converts the measured resistance of the Pt thermistor to temperature and transmits data to a computer. The transcutaneous electro-acupoint stimulator (HANS-200, Nanjing, China) was used to deliver a continuous current stimulation (disperse–dense wave, 2 Hz/100 Hz, 1 mA) during the experiment. As depicted in Figure 9b, during EA, a temperature sensor needle was inserted into the Zusanli point, and a traditional acupuncture needle was inserted into a non-acupoint 5 mm below the acupoint. Both needles were connected to the EA stimulator with metal clips, forming a current loop. Throughout the experiment, the room temperature was controlled at 16 ± 1 °C, and all the rats remained awake.

**Figure 10:**
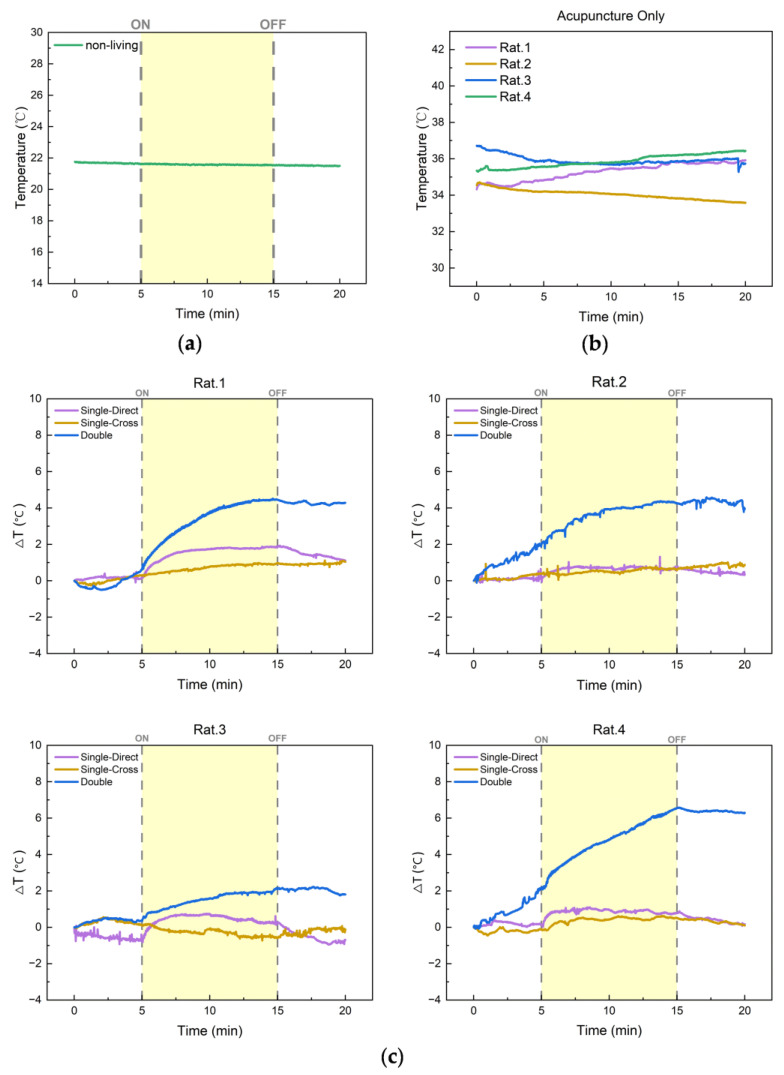
Results of in situ acupoint temperature monitoring in rats. (**a**) EA experiment on a non-living piece of meat. (**b**) Acupuncture only experiments in vivo. (**c**) EA experiments in vivo.

To verify the electrical insulation of the sensor package and to exclude the heat generation caused by EA on non-living flesh tissue, we first performed an EA experiment on a non-living piece of meat at room temperature. As shown in Figure 10a, no temperature change was observed when the EA simulator was turned on or off. This demonstrates that the sensor package is electrically well insulated and EA does not heat non-living flesh tissue.

Keeping the EA simulator off, the temperature response of the Zusanli point was recorded when four rats were stimulated with acupuncture only. As shown in Figure 10b, the temperature fluctuates irregularly which means the acupuncture stimulation does not produce an effect on the temperature of Zusanli points in rats. In other words, this temperature fluctuation only indicates the thermoregulation of the rat itself.

As shown in Figure 9c, EA simulation was applied to Zusanli points of single side and double side, and the temperature sensor needle was used to monitor the direct or cross-temperature response to EA stimulation. The results of these EA experiments in vivo are shown in Figure 10c. In the Single–Direct EA experiment, we observed contractions of rat leg muscles on the EA stimulation side, and a temperature increase at acupoints stimulated by EA, which is consistent with previous studies [11]. In the Single–Cross EA experiment, we did not observe any obvious effect of EA stimulation on the temperature of contralateral acupoints. Comparing the results of these two experiments, muscle contraction is likely the main reason for the acupoint temperature rise. It is well known that muscle contraction consumes adenosine triphosphate (ATP), and as ATP is broken down, some of the energy is released in the form of heat [38]. In the Double EA experiment, after 10 min of EA stimulation, the acupoint temperature increased by 2.92 ± 1.10 °C, which was 2.91 ± 1.09 times higher than that in the Single–Direct EA experiment. Besides muscle contraction, there should be other mechanisms affecting the acupoint temperature that need to be further investigated.

## 5. Conclusions

We have designed a flexible temperature sensor integrated at the needle tip for in situ acupoint temperature monitoring. The temperature sensor has a 125 μm wide by 3.2 cm long flexible structure that can be mass-produced on silicon wafers using MEMS manufacturing technology. The packaged sensor needle exhibits high sensitivity, strong linearity, and excellent repeatability in temperature measurements. In the in vivo acupoint temperature monitoring experiment, we observed a temperature rise at the acupoints stimulated by electro-acupuncture, which can be valuable for acupoint temperature studies.

## Data Availability

The original contributions presented in this study are included in this article; further inquiries can be directed to the corresponding author.

## References

[1] Chen R., Kang M. (2006). Acupoint Heat-Sensitization and Its Clinical Significance. J. Tradit. Chin. Med..

[2] Lingyun G.A.O., Xianglong H.U., Xiaoyang X.U., Baohua W.U., Ling C. (2006). Measurement of the Temperature in Deep Tissues along the Governor Vessel. Acupunct. Res..

[3] Wu Z.-Y., Liu X.-L., Hong W.-X., Zhang D. (2010). Research on the correlation between the temperature asymmetry at acupoints of healthy and affected side and the severity index of facial paralysis. Chin. Acupunct. Moxibustion.

[4] Chen M., Wu Z., Hu X., Xu J. (2011). Effect of Acupuncture on Partial Oxygen Pressure and Temperature of deep tissues along Large Intestine Channel in 30 Normal Volunteer Subfects. Chin. J. Basic Med. Tradit. Chin. Med..

[5] Ma H., Bai X., Wang S., Li S., Tang L., Zhang D. (2013). Influences of electroacupuncture and moxibustion on temperature and blood flow in local tissue. J. Beijing Univ. Tradit. Chin. Med..

[6] Tang L., Wang S., Li S., Zhang D. (2014). Effects of superficial temperature and transcutaneous oxygen pressure of yingxiang point induced by EA Hogu. Chin. J. Basic Med. Tradit. Chin. Med..

[7] Yang Z., Zhou M., Wang X., Zhao Y., Chen Z., Lan Y., Xu T., Zhao Y., Zhao L. (2017). Review on skin temperature of acupoints. Chin. Acupunct. Moxibustion.

[8] Zheng S., Xu J., Pan X., Sa Z., Zhu X., Yang X., Su M., Shen C. (2017). Law of changes in temperature of stomach channel’s acupoints and the effects of electrical acupuncture during gastrointestinal function skewness. China J. Tradit. Chin. Med. Pharm..

[9] Bai H., Ji B., Zhao G., Wang D., Yan M., Sun X., Lu Y., Dai J., Liu Y., Ge Y. (2018). Influence of high frequency electroacupuncture on skin temperature around meridian acupoints, and non-menridian sham acupoints in rats with myocardial ischemia. J. Beijing Univ. Tradit. Chin. Med..

[10] Ma H., Wang S., Song X., Guo M., Wang Y., Li R., Gao R. (2023). Effect of Herbal Patching of Mahuang (Herba Ephedrae) and Huangqin (Radix Scutellariae) at Feishu (BL13) on the Surface Temperature of Neck, Chest and Back Acupoints in Healthy Subjects. J. Tradit. Chin. Med..

[11] Cui R., Liu J., Ma W., Hu J., Zhou X., Li H., Hu J. (2005). A needle temperature microsensor for in vivo and real-time measurement of the temperature in acupoints. Sens. Actuators A Phys..

[12] Tanaka M. (2007). An industrial and applied review of new MEMS devices features. Microelectron. Eng..

[13] Bell D.J., Lu T.J., Fleck N.A., Spearing S.M. (2005). MEMS actuators and sensors: Observations on their performance and selection for purpose. J. Micromechanics Microengineering.

[14] Khan Y., Ostfeld A.E., Lochner C.M., Pierre A., Arias A.C. (2016). Monitoring of Vital Signs with Flexible and Wearable Medical Devices. Adv. Mater..

[15] Lü X.Z., Jiang J.A., Wang H., Gao Q.B., Zhao S.B., Li N., Yang J.Y., Wang S.L., Bao W.M., Chen R.J. (2019). Sensitivity-Compensated Micro-Pressure Flexible Sensor for Aerospace Vehicle. Sensors.

[16] Xie M.Y., Hisano K., Zhu M.Z., Toyoshi T., Pan M., Okada S., Tsutsumi O., Kawamura S., Bowen C. (2019). Flexible Multifunctional Sensors for Wearable and Robotic Applications. Adv. Mater. Technol..

[17] Segev-Bar M., Haick H. (2013). Flexible Sensors Based on Nanoparticles. Acs Nano.

[18] Xiao S.Y., Che L.F., Li X.X., Wang Y.L. (2007). A cost-effective flexible MEMS technique for temperature sensing. Microelectron. J..

[19] Li K.-S., Chao T.-Y., Cheng Y.-T., Chen J.-K., Chen Y.-S. Temperature sensing probe integrated with an SU-8 flexible ribbon cable for heart surgery application. Proceedings of the 16th International Solid-State Sensors, Actuators and Microsystems Conference.

[20] Cui Z., Poblete F.R., Zhu Y. (2019). Tailoring the Temperature Coefficient of Resistance of Silver Nanowire Nanocomposites and their Application as Stretchable Temperature Sensors. Acs Appl. Mater. Interfaces.

[21] Park J.S., Lee D.S., Nho H.W., Kim D.S., Hwang T.H., Lee N.K. (2017). Flexible Platinum Temperature Sensor Embedded in Polyimide Films for Curved Surface Temperature Monitoring Applications: Skin Temperature of Human Body. Sens. Mater..

[22] Jiang H.C., Huang M., Yu Y.B., Tian X.Y., Zhao X.H., Zhang W.L., Zhang J.F., Huang Y.F., Yu K. (2018). Integrated Temperature and Hydrogen Sensors with MEMS Technology. Sensors.

[23] Liu G.F., Chen X.L., Li X.M., Wang C.H., Tian H.M., Chen X.M., Nie B.B., Shao J.Y. (2022). Flexible, Equipment-Wearable Piezoelectric Sensor With Piezoelectricity Calibration Enabled by In-Situ Temperature Self-Sensing. IEEE Trans. Ind. Electron..

[24] He Q.P., Zhang W.Q., Sheng T.Y., Gong Z., Dong Z.H., Zhang D.Y., Jiang Y.G. (2022). Flexible conductivity-temperature-depth-strain (CTDS) sensor based on a CNT/PDMS bottom electrode for underwater sensing. Flex. Print. Electron..

[25] Wang X.Y., Chen X.R., Deng Y., Cheung Y.K., Jiang P., Xu W., Yu H.Y., IEEE A Flexible Thermal Flow Sensor with Quadruple Heaters and Suspended Structure for Performance Enhancement. Proceedings of the 35th IEEE International Conference on Micro Electro Mechanical Systems Conference (IEEE MEMS).

[26] Kreider K.G., Ripple D.C., Kimes A.A. (2009). Thin-film resistance thermometers on silicon wafers. Meas. Sci. Technol..

[27] Riddle J.L., Furukawa G.T., Plumb H.H. (1973). Platinum Resistance Thermometry.

[28] Bass J. (1972). Deviations from Matthiessen’s Rule. Adv. Phys..

[29] Fuchs K. (1938). The conductivity of thin metallic films according to the electron theory of metals. Proc. Camb. Philos. Soc..

[30] Sondheimer E.H. (1952). The mean free path of electrons in metals. Adv. Phys..

[31] Mayadas A.F., Shatzkes M., Janak J.F. (1969). Electrical Resistivity Model for Polycrystalline Films: The Case of Specular Reflection at External Surfaces. Appl. Phys. Lett..

[32] Mayadas A.F., Shatzkes M. (1970). Electrical-Resistivity Model for Polycrystalline Films: The Case of Arbitrary Reflection at External Surfaces. Phys. Rev. B.

[33] Zhang X.G., Butler W.H. (1995). Conductivity of metallic films and multilayers. Phys. Rev. B.

[34] Lacy F. (2011). Developing a theoretical relationship between electrical resistivity, temperature, and film thickness for conductors. Nanoscale Res. Lett..

[35] Vancea J., Hoffmann H., Kastner K. (1984). Mean Free-Path and Effective Density of Conduction Electrons in Polycrystalline Metal-Films. Thin Solid Film..

[36] Kim B.J., Meng E. (2016). Review of polymer MEMS micromachining. J. Micromechanics Microengineering.

[37] Li Z. (2003). Experimental acupuncture science. China Press Tradit. Chin. Med..

[38] Yamada K. (2017). Energetics of muscle contraction: Further trials. J. Physiol. Sci..

